# Trials of improved practices to explore caregivers’ practices for childhood nutrition and acceptance of novel interventions for moderately wasted children in a Dhaka slum

**DOI:** 10.1017/S1368980026102080

**Published:** 2026-02-18

**Authors:** Md Hasan Hafizur Rahman, Ishita Mostafa, Jafrin Ferdous, Kazi Nazmus Saqeeb, Tonmoy Sarkar, Md Musfikur Rahman, Tahmeed Ahmed

**Affiliations:** 1 Nutrition Research Division, International Centre for Diarrhoeal Disease Research, Bangladesh (icddr,b), Dhaka 1212, Bangladesh; 2 Center for Child, Adolescent, and Maternal Health Research, Faculty of Medicine and Health Technology, Tampere University and Tampere University Hospital, Tampere, Finland; 3 Emerging Infections, Infectious Diseases Division, International Centre for Diarrhoeal Disease Research, Bangladesh (icddr,b), Dhaka 1212, Bangladesh; 4 Research and Resource mobilization, Light House, Dhaka, Bangladesh; 5 Office of the Executive Director, International Centre for Diarrhoeal Disease Research, Bangladesh (icddr,b), Dhaka 1212, Bangladesh

**Keywords:** Malnutrition, Trials of Improved Practices, RUSF, MDSF, LAF

## Abstract

**Objective:**

This study used the trials of improved practices (TIPs) approach to explore complementary feeding practices among caregivers of children under two and assess the acceptance of new nutritional supplements by providing microbiota-directed supplementary food (MDSF), ready-to-use supplementary food (RUSF) and locally available food (LAF) among moderately malnourished children.

**Design:**

The study was conducted between May and October 2022 in preparation for a larger trial. The first phase focused on complementary feeding, hygiene, breast-feeding and responsive feeding practices using in-depth interviews and observations. The second phase involved counselling sessions and providing food supplements for forty-five participants. Follow-up visits evaluated acceptability and challenges faced during this period.

**Setting:**

Bauniabadh slum, Mirpur, Dhaka.

**Participants:**

Sixty-five children aged 6–24 months with moderate wasting and their caregivers.

**Results:**

Findings from IDI and observations revealed poor handwashing practices, with most caregivers washing only with water, and inconsistent use of soap. Only a minority boiled drinking water or cleaned utensils with soap. Responsive feeding practices were also limited, with frequent mobile phone use during feeding and lack of attention to the child. Among the three food interventions, LAF received the highest hedonic ratings across all sensory attributes, with a mean taste score of 5·7±1·4, compared with MDSF (4·8±1·9) and RUSF (4·7±1·6), although median consumption was similar across all supplements (75%).

**Conclusion:**

The TIPs approach identified context-specific caregiver behaviours and feeding preferences. These findings will guide the upcoming trial and assist policymakers and program planners in developing culturally tailored interventions to address childhood malnutrition in urban slums.

In many low- and middle-income countries, undernutrition remains a major public health challenge for children under five^(1)^. It manifests in various forms, including stunting, underweight, wasting and micronutrient deficiencies^(1)^ and is closely linked to higher childhood morbidity and accounts for about one-third of deaths in this age group^(2)^. Persistent undernutrition into adulthood increases morbidity and reduces productivity over the lifespan^(2)^. Globally, 47 million under-five children suffer from acute wasting and 149 million from stunting^(3)^. Alarmingly, 35 % of South Asian children under five are stunted, and more than 15 % are wasted (<–2 sd WHZ)^(4)^. In Bangladesh, the prevalence of stunting, underweight and wasting was reported to be 24 %, 22 % and 11 %, respectively^(5)^.

Despite being a sustainable development goal priority, malnutrition remains one of Bangladesh’s most pressing public health issues^(6)^. The issue is especially alarming among the urban underprivileged population in metropolitan areas like Dhaka, where an estimated 5000 slums house millions^(6–8)^. These slums are characterised by a high population density, poor sanitation, limited clean water access, inadequate drainage, frequent diarrhoea outbreaks and pervasive poverty, all negatively affecting children’s health and nutritional status^(9)^. Furthermore, the factors contributing to severe acute malnutrition and moderate acute malnutrition (MAM) are multifaceted and include low birth weight, suboptimal infant and young child feeding practices, limited availability of health services, poor hygiene practices and inadequate access to clean water and sanitation facilities^(2,10–12)^.

Moderate wasting accounts for over 70 % of global cases^(3,13)^ yet receives less programmatic attention than severe wasting. Children with MAM face a 3·1-fold higher mortality risk than well-nourished peers^(3,14)^ and are vulnerable to progressing into severe wasting without timely intervention. This evidence gap hinders cost-effective scale-up of nutrition programs and slows progress towards sustainable development goals^(10,14)^. While comprehensive information exists on managing severe acute malnutrition children, there is limited evidence on the management of children with moderate wasting^(14)^. Recognising this need, the WHO has called for additional evidence to inform cost-efficient interventions and initiated the NUTRIMAM trial (ISRCTN53213318) in three South Asian and two African nations. The idea is to provide three types of food supplements, namely, microbiota-directed supplementary food (MDSF), ready-to-use supplementary food (RUSF) and locally available food (LAF), along with comprehensive nutritional and hygiene-focused education. Nutrition education for mothers can improve knowledge, dietary habits and prevention of illness, and these effects are further enhanced when combined with supplementary meals for managing MAM^(12,15)^. Interventions focused on caregiver education about appropriate complementary feeding have demonstrated significant improvements in nutritional outcomes^(16,17)^. To effectively address the societal context and practical constraints in targeted communities, the program needs to prioritise practices that are both feasible and culturally acceptable^(1,18,19)^.

To evaluate the practicality and acceptability of supplements and counselling of the NUTRIMAM trial, a formative evaluation was conducted using the trials of improved practices (TIPs) approach^(19)^. TIPs is a formative research approach that systematically gathers both quantitative and qualitative data from individuals and communities^(19)^. The data help assess which health behaviours to promote, remove or modify and identify obstacles to adopting new practices such as proper breast-feeding positions, correct handwashing techniques and appropriate complementary feeding^(1)^. TIPs was specifically selected over other formative research methods (e.g. focus groups and cross-sectional surveys) because it uniquely combines real-world practice testing with iterative participant feedback^(18,20)^. Developed by the Manoff Group, TIPs involves a structured series of visits where an interviewer and an interviewee engage in a discussion of current practices, potential areas for improvement and collaboratively arrive at one or a few solutions to implement during a trial period^(18,19)^. This methodology offers a direct avenue for acquiring insights from the participants engaged in the program and also allows them to participate in innovative activities. It also helps researchers identify techniques that align with cultural norms and are acceptable to the community^(21)^. This participatory approach is critical for designing acceptable interventions in resource-constrained settings where prescriptive recommendations often fail^(22)^.

The objective of this TIPs study was to identify key behavioural issues among mothers or caregivers of MAM children, focusing on breast-feeding, complementary feeding, hygiene, feeding practices during illnesses, strategies for managing picky eaters and approaches for delivering the study supplements. It also aimed to explore perceptions regarding packaged food and preparation of study foods at home. The findings were used to tailor the NUTRIMAM trial interventions, particularly by finalising LAF recipes, developing a feeding card to track supplement consumption, developing scripts for counsellors and designing an educational tool to promote behavioural changes at both individual and household levels.

## Methods

### Study setting

This formative study employed a cross-sectional design utilising mixed methods in Bauniabadh slum. Bauniabadh slum, located in Dhaka, Bangladesh, represents the complex challenges faced by urban poor communities. As of a 2014 census, it housed over 34 000 residents across more than 8600 households, with an average household size of four^(23)^. Living conditions are typically precarious; only 39 % of homes are constructed with concrete, while the rest are semi-concrete or temporary shelters^(23)^. Shared sanitation and communal kitchens are the norm, used by over 80 % of residents^(23,24)^. Although piped water is widely available, concerns remain about its quality^(24)^. Health system distrust, environmental hazards including frequent flooding and the risk of fires due to informal electrical wiring further exacerbate vulnerabilities^(24)^.

### Study participants and sampling

Trained health workers first visited households in the study area to identify potential MAM children aged 6–24 months using mid-upper arm circumference (MUAC) tape and a total of 630 children were screened. Of these, 206 had an MUAC of 115–130 mm. We invited the caregivers of these 206 children to visit our field office for further assessment. Among them, 192 attended, and their children’s weight and length were measured to calculate weight-for-length z-score. Based on weight-for-length z-score, eighty-six children were classified as MAM. Enrolment was done using a purposive sampling technique and included a total of sixty-five children diagnosed with MAM (–3 ≤ weight-for-length z-score < –2 or 115 ≤ MUAC < 125 mm) and their mother or primary caregiver. Additionally, we excluded children with severe illnesses, persistent diarrhoea, chronic infections or disabilities requiring hospitalisation or currently receiving food supplements for moderate wasting. Each participant was thoroughly informed about the study and written informed consent was obtained from them.

**Figure 1.**
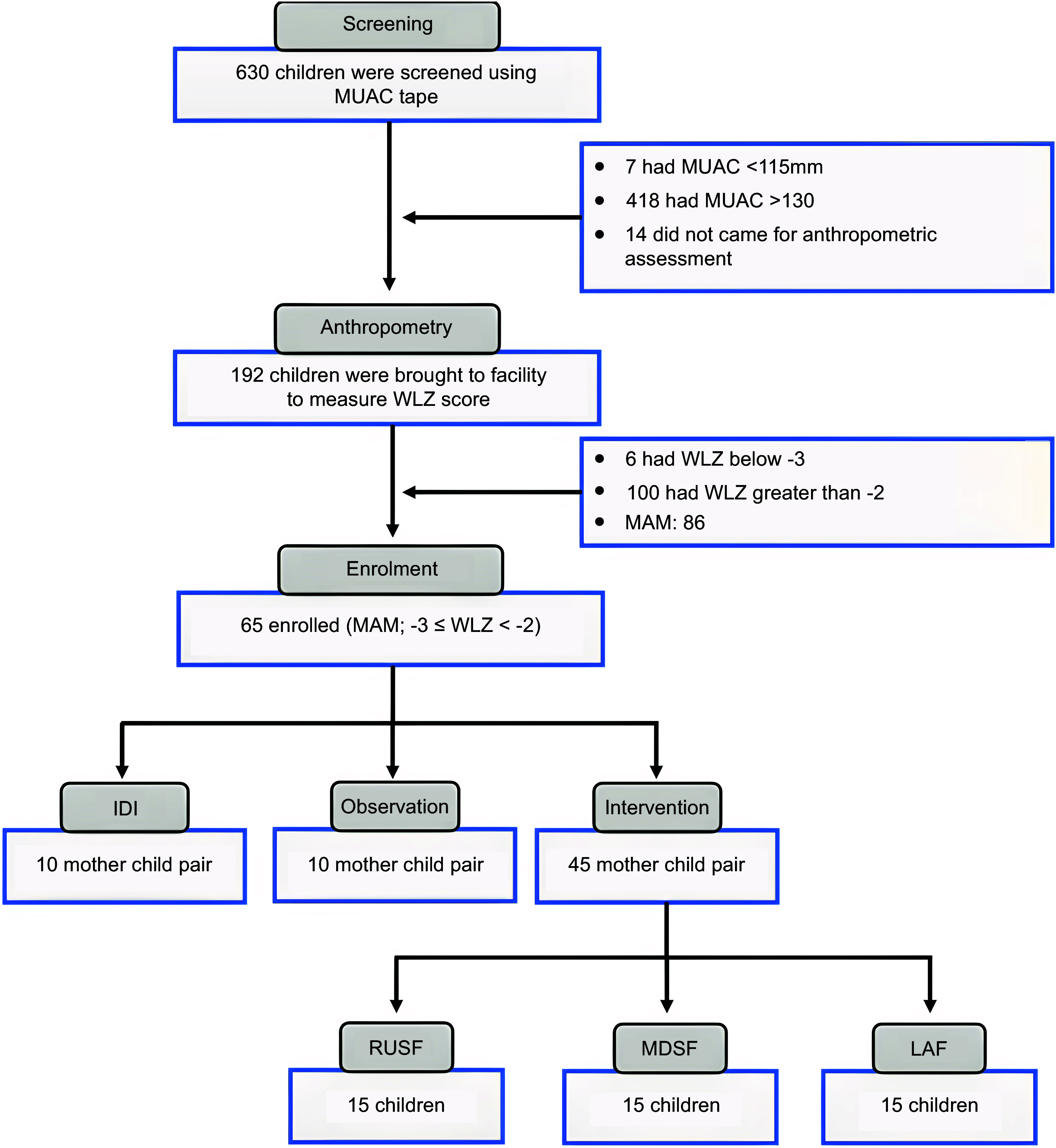
Flow diagram of participant screening, enrolment and intervention allocation.

Among the sixty-five enrolled caregiver-child pairs, ten were purposively selected for IDI and ten for household observations. To reflect diversity in the child’s age, one-third of the children were selected from 6–< 12 months age group, one-third from 12–< 18 months age group and remaining one-third from 18–< 24 months age group. The remaining forty-five participants were allocated to the food intervention arms, stratified by the same age groups to ensure balance. As a formative TIPs study, no power calculation was performed. The sample size was based on methodological guidance emphasising thematic saturation, feasibility and diversity of caregiver perspectives^(25–27)^. The overall flow of screening, enrolment and intervention allocation is illustrated in Figure 1.

### Data collection

We conducted in-depth interviews (IDI) using a semi-structured guide to explore caregivers’ behaviours and challenges related to complementary feeding, breast-feeding, WASH, illness and perceptions of packaged food. We also collected quantitative and anthropometric data using a structured questionnaire. Additionally, we used an observational toolkit to observe and document data on specific behaviours of caregivers at home. To ensure accuracy and reliability of collected data, we provided extensive training for the data collectors covering study objectives, ethics and data collection tools. The interview guideline, observation toolkit and survey questionnaires were adapted from previous studies in similar settings. They were reviewed by the study team and pilot-tested during training and initial field visits. Minor revisions were made based on this pilot feedback.

### Anthropometry

Length and weight were measured by trained personnel using infantometer (0·1 cm precision) and digital scales (2 g sensitivity), and MUAC was assessed with standardised tapes. All measurements were taken twice; if discrepancies exceeded defined cut-offs (MUAC: 0·2 cm, weight: 0·10 kg and length: 0·5 cm), a third measurement was taken and the final value was the average of the two closest. Equipment was routinely calibrated for accuracy.

The whole study was conducted in two phases:

### Phase-1

A team of trained interviewers conducted ten IDI to explore caregivers’ perceptions about hygiene, immunisation, breast-feeding, complementary feeding, feeding during illness and feeding practices for picky eaters. All the IDI were audio-recorded with consent for accurate transcription and analysis. On average, each IDI took 41 min to complete.

To observe daily activities of caregivers, trained field research assistants visited another ten households. They meticulously observed childcare activities for approximately 7 h and documented their observations using the observation toolkit.

### Phase-2

In phase 2, we provided counselling and food interventions to forty-five caregiver–children pairs.

#### Counselling

On the first day, each caregiver received counselling on complementary feeding, breast-feeding, WASH, feeding during illness, feeding techniques for picky eaters and vaccination (Table 1). Additionally, field assistants demonstrated a few practices such as steps for handwashing, breast-feeding attachment and positioning.

**Table 1.** Recommended practices for caregivers of moderately wasted children aged 6–24 months

Hygiene practice Wash hands with soap and water before preparing food, feeding the child and after using toilet Wash the child’s hands before eating Boil drinking water and store in clean jars, regularly clean those jars Wash fruits and vegetables with clean water before giving them to the child Wash hands with soap and water after handling raw meat/fish Wash child’s clothes and bed sheets regularly, drying them under sunlight Maintain cleanliness in the house and surroundings
Food preparation and storage Use clean utensils for food preparation and feeding Wash meat, poultry and fish thoroughly before cooking Serve food immediately after preparation and if needed store safely using solid lids Reheat food before serving if it was prepared 5–6 h earlier
Amount and frequency of feeding Start complementary feeding when the child is 6 months old, gradually increasing the quantity Feed 2 meals a day for 6–8 months, 3 meals a day for 9–11 months and 3 meals plus 1 snack for 12–23 months old child
Food diversity Try to feed a diverse diet from four or more food groups, including cereals, legumes, animal-source foods, fruits and vegetables Avoid low-nutrient-value drinks like soda and sugary juices Provide adequate fat content
Consistency Food consistency should be soft and easy to swallow, not too thick to avoid choking and not too thin to ensure adequate nutrition Avoid choking hazards like nuts, raw carrots, or hard candies
Responsive feeding and feeding practices for picky eaters Feed the child when hungry Provide variety to avoid monotony Do not force-feed Avoid junk food like chips and candy Limit distractions like TV or mobile phones during mealtime Play, sing or tell stories while feeding Avoid scheduling meals close to bedtime
Feeding during illness Continue breast-feeding and increase frequency when the child is sick Feed small, nutritious meals frequently Avoid spicy or oily foods Express breast milk if the child is too weak to suck
Breast-feeding counselling Ensure proper attachment and positioning Use comfortable feeding postures (e.g. cradle hold) Express milk safely if away for long Use expressed milk within 6–8 h at room temperature
General danger signs in children Unable to drink or eat Vomits everything consumed Convulsions or history of convulsions Lethargy or progressive weakness Unconsciousness

#### Food intervention

**Figure 2.**
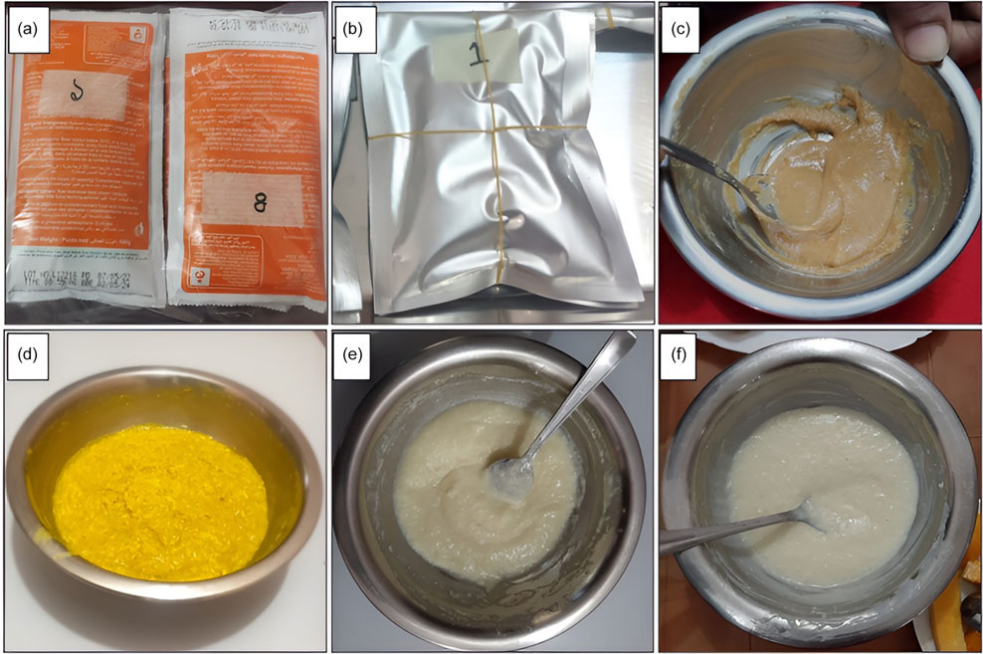
Nutritional supplements provided to study participants. (a) ready-to-use supplementary food (RUSF) sachets. (b) microbiota-directed supplementary food (MDSF) sachets. (c) Prepared MDSF. (d) Prepared Khichuri. (e) Prepared Payesh. (f) Prepared Suji-Firni.

**Figure 3.**
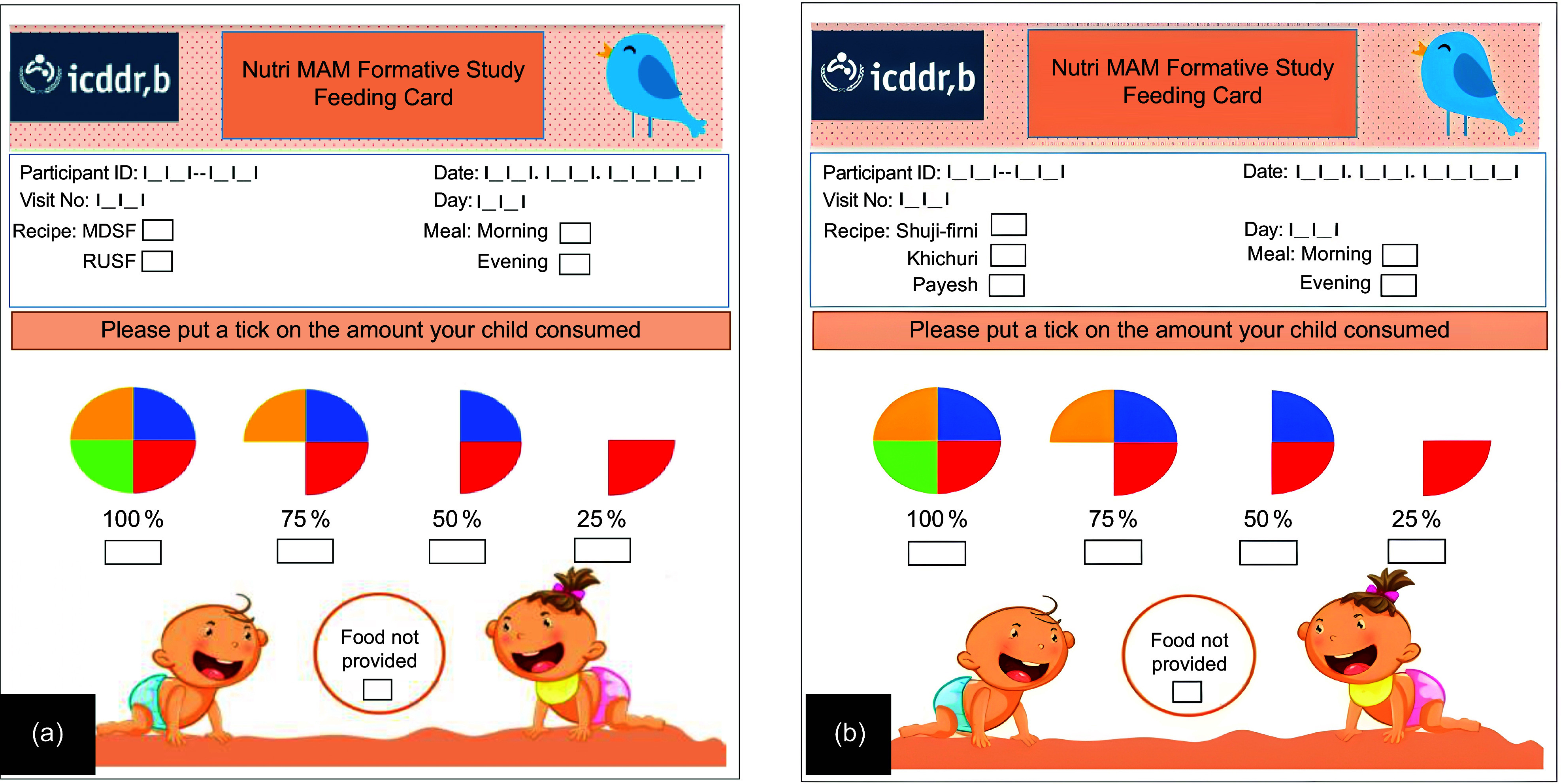
Feeding cards used to assess consumption of the provided supplements. (a) Card for MDSF and RUSF supplements; (b) card for locally available foods (LAF: Suji-Firni, Khichuri, or Payesh). For each meal, caregivers noted the recipe and meal time, and indicated intake by ticking one of five categories: 25 %, 50 %, 75 %, 100 %, or ‘food not provided’. Sections for participant ID, visit number and date were completed by study staff. All feeding cards were provided in the local language (Bangla) to facilitate understanding and consistent reporting. RUSF, ready-to-use supplementary food; MDSF, microbiota-directed supplementary food.

All forty-five participants were divided equally into three intervention arms: MDSF, RUSF and LAF (Figure 2). Each participant received one of the three interventions for 6 days. Our study staff also taught the caregiver how to prepare the food, feed the child and store the leftovers. Every participant was also given a feeding card to be filled out by the mothers to record compliance (Figure 3). Separate feeding cards were provided for each serving of meals for six days. The feeding card was adapted from previous community-based nutrition trials in Bangladesh. Trained staff explained its use to caregivers, and the card was applied consistently across all intervention arms.

For the MDSF arm, each child was offered 100 g per day divided into two separate servings (50 g per serving). Mothers were instructed to finish the first serving within 4–5 h and then offer the second serving.

Fifteen children in the RUSF arm were given one RUSF packet per day for 6 days. Each RUSF packet contained 100 g of supplement, and caregivers were instructed to open a packet in the morning and finish it within 24 h.

For the LAF arm, we provided three different recipes, namely *Khichuri, Payesh* and *Suji-Firni* to all fifteen participants in a crossover design. Each participant received raw ingredients packaged in zip-lock bags, with a ration sufficient for two meals each day. Caregivers were taught how to prepare the recipes at home.

### Follow-up

After the 6-d intervention, follow-up interviews were conducted with all forty-five participants using a semi-structured questionnaire. Responses were obtained and documented regarding their experience of the trial and any obstacles encountered during this period. We also collected consumption data using feeding cards and acceptability data using a seven-point hedonic scale (1 = dislike extremely, 2 = dislike moderately, 3 = dislike slightly, 4 = neither dislike nor like, 5 = like slightly, 6 = like moderately and 7 = like extremely)^(28)^.

### Data analysis

Quantitative data were analysed using R version 4.5.0. Descriptive statistics were presented as medians (interquartile range) or mean (sd) for continuous variables and frequency (percentage) for categorical variables. Group comparisons for continuous data were performed using the One-way ANOVA or Kruskal–Wallis rank-sum test, while categorical data were analysed using Pearson’s *χ* ^2^ test or Fisher’s exact test.

Qualitative data from IDI and observations were analysed using NVivo 12. Transcripts were coded, and related codes were organised into themes. Themes were visually represented in matrices to identify patterns and relationships. Finally, data interpretation explored connections, contradictions and explanations to provide insights into caregivers’ perceptions and practices.

## Results

### Socio-demographic status

The study involved sixty-five children, with a median age of 15·9 months, median weight of 7·3 kg and length of 73·0 cm. Their gender distribution was almost equal (48 % male, 52 % female). Among them, forty came from nuclear families, fourteen from joint families and eleven from extended families. Around 71 % of households were led by fathers, with less than 5 % led by mothers. Approximately 83 % of mothers had some formal education, while fathers generally had slightly higher education levels, with only one being illiterate. Most mothers (92·3 %) were housewives, while others held positions in service, ready-made garments, tailoring or as housemaids. The fathers’ occupational distribution revealed a higher representation in unskilled labour (60 %), followed by skilled workers (38·5 %), with a minimal percentage (1·5 %) reporting unemployment (Table 2).

**Table 2.** Socio-demographic characteristics of children and their primary caregivers or guardians (*n* 65)

Characteristic	Overall		IDI and observation^‡^		Intervention arms^§^ *n* 45						*P*-value^†^
					RUSF		MDSF		LAF		
	*n* 65^*^		*n* 20^*^		*n* 15^*^		*n* 15^*^		*n* 15^*^		
	Median	IQR	Median	IQR	Median	IQR	Median	IQR	Median	IQR	
Age of the child (months)	15·9	11·8–19·6	14·6	11·8–19·2	15·7	11·0–20·1	17·8	12·1–20·6	15·1	11·7–18·9	0·679
Weight of the child (kg)	7·3	6·7–8·0	6·9	6·6–7·9	7·4	6·9–8·1	7·7	7·3–8·1	6·9	6·5–7·8	0·265
Length of the child (cm)	73·0	69·0–77·2	71·4	68·3–76·7	73·0	68·9–77·5	74·5	72·9–78·1	72·5	68·3–77·1	0·348
Age of the mother (years)	25·0	23·0–30·0	25·5	23·0–31·5	24·0	22·0–30·0	25·0	23·0–32·0	23·0	21·0–29·0	0·485
	***n***	**%**	***n***	**%**	***n***	**%**	***n***	**%**	***n***	**%**	
Sex of the children											0·967
Male	31	48 %	9	45 %	8	53 %	7	47 %	7	47 %	
Female	34	52 %	11	55 %	7	47 %	8	53 %	8	53 %	
Mother’s education											0·766
Formal education	54	83 %	18	90 %	13	87 %	12	80 %	11	73 %	
Only can write name	10	15 %	2	10 %	2	13 %	3	20 %	3	20 %	
Cannot write name	1	1·5 %	0	0 %	0	0 %	0	0 %	1	6·7 %	
Mother’s occupation											0·300
Housewife	60	92·3 %	19	95 %	12	80 %	15	100 %	14	93 %	
Employed	5	7·7 %	1	5·0 %	3	20 %	0	0 %	1	6·7 %	
Father’s education											0·978
Formal education	47	72 %	15	75 %	11	73 %	10	67 %	11	73 %	
Only can write name	17	26 %	5	25 %	4	27 %	4	27 %	4	27 %	
Cannot write name	1	1·5 %	0	0 %	0	0 %	1	6·7 %	0	0 %	
Father’s occupation											0·385
Skilled worker	25	38·5 %	11	55 %	4	27 %	5	33 %	5	33 %	
Unskilled worker	39	60 %	9	45 %	10	67 %	10	67 %	10	67 %	
Unemployed	1	1·5 %	0	0 %	1	6·7 %	0	0 %	0	0 %	
Household head											0·690
Mother	3	4·6 %	1	5·0 %	0	0 %	0	0 %	2	13 %	
Father	46	71 %	14	70 %	13	87 %	11	73 %	8	53 %	
Grandmother	10	15 %	4	20 %	1	6·7 %	2	13 %	3	20 %	
Grandfather	6	9·2 %	1	5·0 %	1	6·7 %	2	13 %	2	13 %	
Type of Family											<0_·_001
Nuclear	40	62 %	15	75 %	10	67 %	9	60 %	6	40 %	
Joint	14	22 %	3	15 %	2	13 %	0	0 %	9	60 %	
Extended	11	17 %	2	10 %	3	20 %	6	40 %	0	0 %	

IDI, in-depth interview; RUSF, ready-to-use supplementary food; MDSF, microbiota-directed supplementary food; LAF, locally available food; IQR, interquartile range.

* Median (IQR); *n* (%).

† Kruskal–Wallis rank sum test; Pearson’s *χ* ^2^ test; Fisher’s exact test.

‡ IDI and Observation (*n* 20) represent participants included in qualitative assessments.

§ RUSF, MDSF and LAF arms (*n* 45, 15 per arm) represent participants tested for acceptability of supplementary foods.

### Findings from in-depth interview and observation

Findings from the initial phase of the study, which included IDI with ten caregivers and structured household observations of 10 caregiver–child pairs, revealed several key issues related to hygiene, breast-feeding and feeding practices. These qualitative insights are presented below.

#### Knowledge, attitude and practice related to safe drinking water in the community

Most families relied on scheduled water supply (1–3 times daily) or deep tube wells for drinking water. While many mentioned boiling and storing water, gas shortages often prevented them from doing so. Observations revealed that most households did not boil water or use other purification methods. Despite this, most respondents understood the importance of safe drinking water, stating that waterborne diseases such as diarrhoea, cholera or dysentery could occur if they drank untreated water.

*‘Recently my husband and children suffered from stomach pain and diarrhoea. If we did not drink boiled water, this problem would be more frequent. I always try to boil water for my family members, but it’s challenging due to insufficient gas supply’* (40-year-old housewife with an 18-month-old child).

#### Knowledge, attitude and practice related to handwashing, sanitation and hygiene practices

All participants recognised the importance of handwashing with soap to prevent illness. They reported washing hands before meals, while feeding children, after using the toilet and after cleaning their child’s stool. However, while soap and water were generally available, most lacked knowledge of proper handwashing techniques or duration. Observations revealed gaps between reported and actual practices, particularly before food preparation and after handling raw meat or fish and sometimes after cleaning a child’s perineal area.

Regarding sanitation, shared toilets were common. Adults consistently used sanitary latrines, while young children typically used plastic commodes (‘potties’). Mothers usually disposed of this waste in toilets or near open sewer lines. For food hygiene, participants reported washing vegetables before cooking, covering food and cleaning utensils with soap and water. Yet observations showed these practices were inconsistent; some mothers used water only and utensils were not always clean before feeding children.

#### Mothers’ knowledge and attitude about breast-feeding

Participants were aware of the benefits of breast-feeding for child development. They said it strengthens the child and supports their mental and physical growth. Some mothers also mentioned health benefits for themselves, such as preventing breast pain and fever.

*‘If I breastfeed my child, he will be intelligent. Breast milk means nutrition for him. Whatever I am eating, he is getting that nutrition through breast milk. He can’t eat like an adult, so breast milk benefits him. I also fed my child colostrum just after his birth’* (27-year-old mother of a 9-month-old child).

A common misconception was that breast milk alone meets all nutritional needs, leading them to prioritise breast-feeding over complementary feeding even after the recommended age. This perspective was echoed by a mother of a 17 month old:

*‘I am breastfeeding my child. It will enhance her physical and mental growth. She will be intelligent, and it will protect her from any diseases. Many people asked me to feed her other food. But I didn’t provide her complementary food because breast milk is enough for her. As much as I can, I will feed her breastmilk. I will give her other food if she does not get breast milk’* (Housewife, age: 27 years).

Conflicting views emerged regarding optimal breast-feeding duration by gender. Some mothers believed boys and girls should be breastfed for the same period, while others expressed the opinion that boys should be breastfed longer than girls, or vice versa. The most common responses suggested 2·5 years for boys and 2 years for girls, while some believed 2 years for boys and 3 years for girls.

*‘Our guardians told us that boys should leave the breast milk earlier, and girls should breastfeed for at least up to three years’* (25-year-old housewife having a 19-month-old child).

In contrast, another mother of a 15-month-old boy said:

*‘A boy will be a man in later life. They will do more work than the girls in the future life. He needs more nutrition. Girls also need nutrition, but boys require more. So, boys should breastfeed more than girls’* (Housewife, age: 22 years).

#### Mothers’ practice related to breast-feeding

Breast-feeding frequency varied widely among children, ranging from 1–2 to 15–16 times daily. Mothers dedicated time exclusively for breast-feeding, with each session lasting 10–15 min up to half an hour, though household responsibilities sometimes shortened these sessions. A 23-year-old mother stated that,

*‘I used to give enough time to my child during breastfeeding. I do not interrupt her if I do not have much work. I would have even spent 30 min to one hour if I were not too busy. But I usually spend 10 to 15 min breastfeeding if I have other household work. If I were not busy, breastfeeding time is a small leisure time for me also’* (Housewife, having a child of 8 months).

Most mothers preferred lying positions for breast-feeding but sometimes adapted the position to what their child preferred. Another mother of a 21-month-old child stated that,

*‘My child preferred both positions. When asleep, he prefers to drink breast milk lying down; when he does not, he prefers to drink sitting. It’s up to him. I always breastfeed him in his preferred position’* (Housewife, 25 years).

However, no participant could describe standard breast-feeding positions, head support techniques or proper nipple alignment. Observations reflected these knowledge gaps, as feeding positions and attachment consistently deviated from recommended practices, and mothers frequently demonstrated limited patience during feeding.

#### Initiation of complementary feeding

Most mothers introduced complementary foods after 6 months, typically starting with formula or powdered milk while continuing breast-feeding. Homemade foods soon became the norm, with semolina (suji), noodles and bananas as initial choices. Within 2–3 months, children were introduced to family foods such as soft hotchpotch or vegetable blends. Diets gradually diversified to include rice, vegetables, eggs, cow’s milk, lentils and commercial foods like noodles or biscuits. A minority of mothers (20 %) relied on formula milk or commercial feeds for a longer period.

One mother stated about the feeding practices that,

*‘We usually eat three times a day. I tried to feed him with me. I gave him snacks between these three meals, like bananas, biscuits, or bread. Due to a gas supply shortage, I couldn’t prepare homemade snacks for him’* (a 21-year-old housewife and mother of a 10-month-old child).

#### Feeding practices for children

Most mothers encouraged self-feeding and allowed sufficient mealtime, typically avoiding force-feeding. Some engaged their children through talking, playing or walking during feeding sessions. A 40-year-old mother stated that,

*‘I never forced my children to eat. If he didn’t want to eat, I tried two or three times, then left the food; sometimes, I kept the food in front of him, and if he liked to eat something, he could. Otherwise, I left the food. I heard forcing is not good, and I never did that’* (Housewife having a child of 18 months old).

However, opposite practices also existed in the community. Some mothers force their children to eat food. One mother admitted:

*‘Sometimes I forced my child. She needs to eat some food. If she didn’t eat food, I had to force her. Sometimes I took her outside of the home, sometimes to the roof. Sometimes if she didn’t want to eat, I beat her’* (a garment worker, age: 22 years, mother of a 15-month-old child).

Observations revealed that half of the mothers showed inadequate attention and patience during feeding. Responsive feeding was often lacking with distractions such as mobile phones. Some mothers resorted to force-feeding, without offering an alternative when children refused. If children showed reluctance to eat homemade food, mothers frequently offered commercially prepared alternatives including chocolate, chips, biscuits, cake, bread and noodles. These were usually consumed in the afternoon or evening, replacing dinner.

#### Feeding during illness

During illness, children commonly reduced their food intake and were reluctant to eat family meals or breastmilk. Mothers reported that their children commonly preferred outside foods during sickness, and they tried to offer whatever foods their children liked. In cases of diarrhoea, all mothers were aware of and practiced administering Oral Rehydration Solution.

### Findings of follow-up visit

#### Handwashing

Most mothers reported washing hands before preparing food, feeding their child and after using the toilet or cleaning the child. Many felt cleaner after following our instructions to wash with soap and water. However, a few struggled due to time constraints, limited soap or water or forgetfulness. Most wished to continue these practices and educate neighbours or relatives, though some were concerned that others might not take them seriously because of social stigma.

#### Breast-feeding

Almost all mothers understood the recommendation to breastfeed their child up to 2 years, regardless of sex, while following proper techniques. Those who tried the new positions reported greater comfort for themselves and their children and intended to continue in the future.

#### Responsive feeding

The majority of caregivers practiced responsive feeding by talking, singing or pointing out objects, while avoiding force-feeding and digital distractions. When children refused food, they stored leftovers in closed containers for later use after reheating. Although most successfully boiled drinking water, some faced obstacles such as interruptions in gas or electricity supply during boiling or reheating.

#### Intervention

Consumption patterns showed that LAF was generally consumed at slightly higher rates than RUSF and MDSF. Overall compliance over the 6-d intervention was good, with mean consumption of 68·9 % for LAF, 68·1 % for RUSF and 62·5 % for MDSF (Table 3). Among the LAF recipes, *Suji-Firni* was particularly convenient due to its short preparation time (5–8 min), while *Khichuri* and *Payesh* required more time.

**Table 3.** Consumption (%) of three supplementary foods: RUSF, MDSF and LAF, presented as mean (sd) and median (IQR)

Characteristic	Overall		RUSF		MDSF		LAF		
	*n* 45		*n* 15		*n* 15		*n* 15		*P*-value
Consumption (%) – Mean (sd)	66·2	33·4	68·1	31·3	62·5	38·8	68·9	27·7	0·400^*^
Consumption (%) – Median (IQR)	75·0	25–100	75·0	25–100	75·0	25–100	75·0	50–100	0·727^†^

RUSF, ready-to-use supplementary food; MDSF, microbiota-directed supplementary food; LAF, locally available food; IQR, interquartile range.

* One-way ANOVA.

† Kruskal–Wallis rank sum test.

Based on sensory evaluation, mean scores for colour, taste, aroma and appetite were generally favourable across all three supplementary foods (Table 4). LAF scored slightly higher than RUSF and MDSF in all categories, with the highest ratings for colour (5·8 (sd 1·3)) and taste (5·7 (sd 1·4)). A statistically significant difference was observed for colour (*P* = 0·048), while differences in taste, aroma and appetite were NS. A few mothers noted that the sweetness level was slightly higher than their regular diet, and there was unfamiliarity with packaged foods. Additionally, the sticky texture sometimes caused the food to adhere to the upper palate of the children.

**Table 4.** Sensory evaluation scores (mean (sd)) provided by caregivers for three supplementary foods: RUSF, MDSF and LAF

	Overall		RUSF		MDSF		LAF		
	*n* 45*		*n* 15*		*n* 15*		*n* 15*		
	Mean	sd	Mean	sd	Mean	sd	Mean	sd	*P*-value^†^
Characteristic									
Colour	5·4	1·6	4·8	1·7	4·8	1·9	5·8	1·3	0·048
Taste	5·3	1·6	4·7	1·6	4·8	1·9	5·7	1·4	0·058
Aroma	5·4	1·4	4·9	1·6	5·3	1·1	5·6	1·4	0·323
Appetite	4·5	0·9	4·5	1·3	4·2	0·6	4·6	0·9	0·185

RUSF, ready-to-use supplementary food; MDSF, microbiota-directed supplementary food; LAF, locally available food.

* Mean (sd).

† One-way ANOVA.

Approximately half of the mothers preferred weekly home visits for interventions, while others favoured intervals of 3–4 d between each delivery. Two-thirds felt that all counselling topics could be covered in a single, longer session, though they recognised the difficulty of retaining and applying all information at once. The remaining third recommended shorter, separate sessions focused on specific topics to improve message retention and practical implementation.

## Discussion

Formative research using the TIPs method is valuable in the development of behavioural programs as it provides a comprehensive understanding of contextual factors and the current habits and beliefs of community members who might benefit from the program. This understanding is crucial for designing effective programs and predicting their impact on nutrition-related outcomes^(1,22,29–33)^. The TIPs process outlined in this article aimed to validate the feasibility of the planned intervention and counselling prior to implementing the ‘NUTRIMAM’ trial. The proposed food interventions were assessed in households, and messages were adjusted during follow-up visits. This study explored caregivers’ usual practices and the barriers they faced when suggested to improve these practices. We also sought to understand their reactions when offered new packaged foods and alternative ways of cooking for their child using locally available ingredients. The process provided additional and often unexpected insights regarding hygiene and feeding practices among caregivers in this context.

The study underscores the significance of adopting safe water practices and the challenges caregivers face in maintaining consistent access to clean water. Despite awareness of waterborne diseases, limited access to resources, such as gas for boiling water, remains a significant barrier. Results from previous research that assessed slum dwellers’ access to safe drinking water in Dhaka appear to corroborate these findings^(34)^. While most caregivers understood the importance of handwashing, a gap between knowledge and observed practice was evident. This discrepancy mirrors global trends where the adoption of handwashing remains limited, despite advocacy efforts in Bangladesh and other regions to promote this crucial technique for disease prevention^(35–38)^.

Misconceptions about breast-feeding duration and exclusivity highlight the need for targeted education. There is a necessity to emphasise the complementary nature of breast-feeding and the proper introduction of complementary feeding after 6 months. Observations and interviews also revealed varied complementary feeding practices, limited dietary diversity and gaps in responsive caregiving. Similar findings have been reported among mothers of infants aged 6–24 months in Dhaka slums^(39)^.

The trial of different food supplements, including MDSF, RUSF and LAF, presents nuanced insights into their acceptability and the challenges faced by caregivers. The intervention highlighted generally good acceptance of all three supplementary foods, though practical challenges were noted. Slightly lower intake for some children may reflect the short duration of the intervention and the adaptation period required for new foods or minor illness episodes. Consumption of MDSF and RUSF may potentially be affected by barriers such as unfamiliarity with packaged foods, the sticky texture or more sweetness than their usual diet. Addressing these issues might involve diluting a small portion of MDSF or RUSF with milk or water just before feeding, particularly for children transitioning from exclusive breast-feeding. A previous study also suggested that therapeutic foods be mixed with other ingredients to improve taste and swallowability^(40)^. LAF recipes, particularly *Suji-Firni*, were more convenient due to shorter preparation times, and higher acceptance of LAF may be attributed to its familiarity, as it uses ingredients already part of children’s regular diets. Moreover, similar sensory scores across all attributes for the three interventions suggest none had undesirable effects, supporting their integration into routine feeding practices.

To enhance the acceptability of MDSF and RUSF, we propose peer-led demonstrations by early adopters, use of single-serving sachets, culturally appropriate packaging and tailored counselling^(40–43)^. These approaches address barriers related to preparation burden, unfamiliar taste, and distrust of packaged foods and align with successful strategies in similar settings. In contrast, the positive reception of LAF recipes signifies the feasibility of utilising locally available familiar ingredients for scaled-up program, though implementation requires addressing key operational barriers^(19)^. Evidence from TIPs studies in Uganda shows that caregivers are more likely to adopt recipes that use familiar ingredients and preparation methods^(19)^. While LAF’s cultural acceptability and caregiver familiarity support adoption, successful scale-up may involve simplifying preparation through pre-mixed ingredient kits^(15)^, introducing community-based production hubs to mitigate shortages of utilities (gas or water)^(34)^ and integrating LAF distribution with existing health service platforms like vaccination campaigns^(44)^. These adaptations reflect time‑saving community feeding strategies that have proven effective in similar contexts^(19)^. However, decentralised production requires strong quality control to prevent nutrient loss and safety issues^(45)^. Importantly, cost-effectiveness and supply-chain feasibility of LAF *v*. packaged supplements will be evaluated in the upcoming NUTRIMAM trial. These assessments will inform sustainability and scalability.

The study was conducted in a single urban slum, and findings may not be generalisable to other slum or rural settings with different socio-economic or cultural contexts; therefore, multi-site replications across diverse settings are needed. Second, the one-week trial period for new techniques, while aligned with the TIPs manual, may be insufficient to assess sustained behaviour change. Increasing the length of this study, as has been shown in earlier studies^(20,46)^ and as planned for NUTRIMAM trial, may help assess sustained behaviour change more effectively. Third, feeding practices were assessed through caregiver-reported cards, which are vulnerable to recall and social desirability bias. Direct methods like supplement weighing or observed feeding would strengthen validity. Fourth, unmeasured confounders such as maternal education or prior exposure to nutrition interventions, seasonal income changes, food insecurity or other concurrent health interventions may have influenced the outcomes but were not captured and should be considered in future trials. Occasionally, caregivers may provide information that aligns with societal expectations rather than their actual experiences or intentions, thereby influencing our interpretation of the study findings. Additionally, presence of observers during household visits may have influenced caregiver behaviour, a phenomenon known as the Hawthorne effect^(47,48)^. Although the team aimed to minimise reactivity through rapport-building, this potential observer bias is a recognised limitation in behavioural studies^(48)^. Lastly, as a formative, exploratory study, it was not powered for statistical comparisons or hypothesis testing. Despite these limitations, these constraints do not undermine the significance of these findings as fundamental information to shape future interventions or programs.

### Conclusion

This mixed-methods TIPs study identified critical context-specific behaviours and preferences affecting feeding practices among caregivers of moderately wasted children in a Dhaka slum. Caregivers reported suboptimal hygiene practices, rarely boiling drinking water and washing hands with water only, and using mobile phones during feeding, reducing responsive engagement. In the supplement trial, all three interventions (MDSF, RUSF and LAF) achieved similar median consumption, but caregivers consistently rated the LAF recipes higher in taste and overall appeal than MDSF or RUSF. These findings suggest that while all supplements were generally acceptable, familiar, locally sourced foods may improve adherence.

Immediate next steps include finalising simplified LAF recipes for household and community kitchens and developing a shelf-stable version of MDSF. Training modules should be developed for community health workers to deliver supplement-specific counselling and reinforce hygiene and feeding practices. Concurrently, policy-level action is needed, requiring multi-stakeholder taskforces involving government, NGO and manufacturers to address systemic barriers, including intermittent utilities and food access challenges, through coordinated community nutrition strategies. Future research should evaluate the cost-effectiveness of these approaches across diverse South Asian contexts while monitoring long-term impacts on child nutrition, growth and development. These steps will help translate formative research insights into sustainable interventions that improve child nutrition and caregiving practices in urban slum settings.

## References

[1] Williams PA , Schnefke CH , Flax VL et al. (2019) Using trials of improved practices to identify practices to address the double burden of malnutrition among Rwandan children. Public Health Nutr 22, 3175–3186. 31221234 10.1017/S1368980019001551 PMC10260626

[2] Rahman A , Bhuiyan MB & Das SK (2022) Effect of short-term educational intervention on complementary feeding index among infants in rural Bangladesh: a randomized control trial. BMC Nutr 8, 73. 35918734 10.1186/s40795-022-00565-0 PMC9344650

[3] Mostafa I , Fahim SM , Das S et al. (2022) Developing shelf-stable Microbiota Directed Complementary Food (MDCF) prototypes for malnourished children: study protocol for a randomized, single-blinded, clinical study. BMC Pediatr 22, 385. 35778675 10.1186/s12887-022-03436-6 PMC9247958

[4] Wali N , Agho KE & Renzaho A (2021) Wasting and associated factors among children under 5 years in five South Asian Countries (2014–2018): analysis of demographic health surveys. Int J Environ Res Public Health 18, 4578. 33925898 10.3390/ijerph18094578 PMC8123503

[5] BDHS (2022) Bangladesh Demographic and Health Survey. https://www.dhsprogram.com/pubs/pdf/FR386/FR386.pdf (accessed November 2024).

[6] Rana Z , Alam M , Islam S et al. (2020) Undernutrition and dietary diversity of children and mothers living in poor urban settings in Dhaka, Bangladesh: a cross-sectional study. J Popul. Dev. 2, 68–83.

[7] Ahmed I (2016) Building resilience of urban slums in Dhaka, Bangladesh. Procedia – Soc Behav Sci 218, 202–213.

[8] Gruebner O , Sachs J , Nockert A et al. (2014) Mapping the slums of Dhaka from 2006 to 2010. Dataset Pap Sci 2014, e172182.

[9] Uddin J , Koehlmoos TP , Saha NC et al. (2012) Strategies for providing healthcare services to street-dwellers in Dhaka city: evidence from an operations research. Health Res Policy Syst 10, 19. 22694892 10.1186/1478-4505-10-19 PMC3536682

[10] Islam MR , Rahman MS , Rahman MM et al. (2020) Reducing childhood malnutrition in Bangladesh: the importance of addressing socio-economic inequalities. Public Health Nutr 23, 72–82. 31203835 10.1017/S136898001900140X PMC10200495

[11] Ahmed T & Ahmed AMS (2009) Reducing the burden of malnutrition in Bangladesh. BMJ 339, b4490–b4490. 19889736 10.1136/bmj.b4490

[12] Kajjura RB , Veldman FJ & Kassier SM (2019) Effect of nutrition education on knowledge, complementary feeding, and hygiene practices of mothers with moderate acutely malnourished children in Uganda. Food Nutr Bull 40, 221–230. 31067997 10.1177/0379572119840214

[13] UNICEF, WHO & World Bank (2021) The UNICEF/WHO/WB Joint Child Malnutrition Estimates (JME) Group Released New Data for 2021. https://www.who.int/news/item/06-05-2021-the-unicef-who-wb-joint-child-malnutrition-estimates-group-released-new-data-for-2021 (accessed April 2022).

[14] WHO, FAO, UNICEF et al. (2020) Global Action Plan on Child Wasting a Framework for Action to Accelerate Progress in Preventing and Managing Child Wasting and the Achievement of the Sustainable Development Goals. https://www.unicef.org/media/96991/file/Global-Action-Plan-on-Child-Wasting.pdf (accessed November 2024).

[15] Lassi ZS , Das JK , Zahid G et al. (2013) Impact of education and provision of complementary feeding on growth and morbidity in children less than 2 years of age in developing countries: a systematic review. BMC Public Health 13, S13. 24564534 10.1186/1471-2458-13-S3-S13 PMC3847349

[16] Bhandari N , Mazumder S , Bhan MK et al. (2004) An educational intervention to promote appropriate complementary feeding practices and physical growth in infants and young children in rural Haryana, India. J Nutr 134, 2342–2348. 15333726 10.1093/jn/134.9.2342

[17] Santos I , Victora CG , Martines J et al. (2001) Nutrition counseling increases weight gain among Brazilian children. J Nutr 131, 2866–2873. 11694610 10.1093/jn/131.11.2866

[18] MANOFF-GROUP (2005) Trials of Improved Practices (TIPs) Giving Participants a Voice in Program Design. https://www.behaviourchange.net/docs/trials-of-improved-practices-tips.pdf (accessed November 2024).

[19] Bekele H & Turyashemererwa F (2019) Feasibility and acceptability of food-based complementary feeding recommendations using trials of improved practices among poor families in rural Eastern and Western Uganda. Food Sci Nutr 7, 1311–1327. 31024704 10.1002/fsn3.964 PMC6475803

[20] Shivalli S , Srivastava RK & Singh GP (2015) Trials of Improved Practices (TIPs) to enhance the dietary and iron-folate intake during pregnancy- a quasi experimental study among rural pregnant women of Varanasi, India. PLOS ONE 10, e0137735. 26367775 10.1371/journal.pone.0137735 PMC4569533

[21] Dickin K , Griffiths M & Piwoz E (1997) Designing by Dialogue: A Program Planners’ Guide to Consultative Research for Improving Young Child Feeding. https://www.eldis.org/document/A27958 (accessed August 2023).

[22] Bentley ME , Johnson SL , Wasser H et al. (2014) Formative research methods for designing culturally appropriate, integrated child nutrition and development interventions: an overview. Ann N Y Acad Sci 1308, 54–67. 24673167 10.1111/nyas.12290 PMC4269231

[23] Khalequzzaman Md , Chiang C , Hoque BA et al. (2017) Population profile and residential environment of an urban poor community in Dhaka, Bangladesh. Environ Health Prev Med 22, 1. 29165111 10.1186/s12199-017-0610-2 PMC5661908

[24] Hasan MZ , Rabbani MG , Ahmed MW et al. (2024) Assessment of socioeconomic and health vulnerability among urban slum dwellers in Bangladesh: a cross-sectional study. BMC Public Health 24, 2946. 39448982 10.1186/s12889-024-20425-9 PMC11515451

[25] Hennink M & Kaiser BN (2022) Sample sizes for saturation in qualitative research: a systematic review of empirical tests. Soc Sci Med 292, 114523. 34785096 10.1016/j.socscimed.2021.114523

[26] Pelto GH & Armar-Klemesu M (2011) Balancing nurturance, cost and time: complementary feeding in Accra, Ghana. Matern Child Nutr 7, 66–81. 21929636 10.1111/j.1740-8709.2011.00351.x PMC6860681

[27] Sharma SK , Mudgal SK , Gaur R et al. (2024) Navigating sample size estimation for qualitative research. J Med Evid 5, 133.

[28] Sharif M , Butt M , Sharif H et al. (2017) Sensory evaluation and consumer acceptability. In Handbook of Food Science and Technology, pp. 362–386 [ IA Khan , M Farooq , editors]. Faisalabad, Pakistan: University of Agriculture.

[29] Daelmans B , Ferguson E , Lutter CK et al. (2013) Designing appropriate complementary feeding recommendations: tools for programmatic action. Matern Child Nutr 9, 116–130. 24074322 10.1111/mcn.12083 PMC6860844

[30] Fabrizio CS , Van Liere M & Pelto G (2014) Identifying determinants of effective complementary feeding behaviour change interventions in developing countries. Matern Child Nutr 10, 575–592. 24798264 10.1111/mcn.12119 PMC4282339

[31] Paul KH , Muti M , Khalfan SS et al. (2011) Beyond food insecurity: how context can improve complementary feeding interventions. Food Nutr Bull 32, 244–253. 22073798 10.1177/156482651103200308

[32] Pelto GH , Levitt E & Thairu L (2003) Improving feeding practices: current patterns, common constraints, and the design of interventions. Food Nutr Bull 24, 45–82. 12664527 10.1177/156482650302400104

[33] Sanghvi T , Jimerson A , Hajeebhoy N et al. (2013) Tailoring communication strategies to improve infant and young child feeding practices in different country settings. Food Nutr Bull 34, S169–S180. 24261075 10.1177/15648265130343S204

[34] Haque SS , Yanez-Pagans M , Arias-Granada Y et al. (2020) Water and sanitation in Dhaka slums: access, quality, and informality in service provision. Water Int 45, 791–811.

[35] Curtis VA , Danquah LO & Aunger RV (2009) Planned, motivated and habitual hygiene behaviour: an eleven country review. Health Educ Res 24, 655–673. 19286894 10.1093/her/cyp002 PMC2706491

[36] Luby SP , Kadir MA , Yushuf Sharker MA et al. (2010) A community-randomised controlled trial promoting waterless hand sanitizer and handwashing with soap, Dhaka, Bangladesh. Trop Med Int Health TM IH 15, 1508–1516. 20958896 10.1111/j.1365-3156.2010.02648.x

[37] Freeman MC , Stocks ME , Cumming O et al. (2014) Hygiene and health: systematic review of handwashing practices worldwide and update of health effects. Trop Med Int Health TM IH 19, 906–916. 24889816 10.1111/tmi.12339

[38] Amin N , Sagerman DD , Nizame FA et al. (2019) Effects of complexity of handwashing instructions on handwashing procedure replication in low-income urban slums in Bangladesh: a randomized non-inferiority field trial. J Water Sanit Hyg Dev 9, 416–428.

[39] Saleh F , Ara F , Hoque MdA et al. (2014) Complementary feeding practices among mothers in selected slums of Dhaka city: a descriptive study. J Health Popul Nutr 32, 89–96. 24847597 PMC4089076

[40] Nikièma V , Fogny NF , Kangas ST et al. (2022) Availability, use, and consumption practices of ready-to-use therapeutic foods prescribed to children with uncomplicated severe acute malnutrition aged 6–59 months during outpatient treatment in Burkina Faso. Appetite 168, 105751. 34648913 10.1016/j.appet.2021.105751

[41] Haque NB , Mihrshahi S & Haider R (2023) Peer counselling as an approach to improve complementary feeding practices: a narrative review. J Health Popul Nutr 42, 60. 37403126 10.1186/s41043-023-00408-z PMC10320908

[42] Ara G , Khanam M , Papri N et al. (2019) Peer counseling promotes appropriate infant feeding practices and improves infant growth and development in an urban slum in Bangladesh: a community-based cluster randomized controlled trial. Curr Dev Nutr 3, nzz072. 31334480 10.1093/cdn/nzz072 PMC6635820

[43] Ahmed T , Choudhury N , Hossain MI et al. (2014) Development and acceptability testing of ready-to-use supplementary food made from locally available food ingredients in Bangladesh. BMC Pediatr 14, 164. 24972632 10.1186/1471-2431-14-164 PMC4098698

[44] Lanou HB , Somé JW , Koumbem MAA et al. (2025) Microbiome-directed food to promote sustained recovery in children with uncomplicated acute malnutrition: protocol for a randomized controlled trial in Burkina Faso. BMC Nutr 11, 92. 40361242 10.1186/s40795-025-01045-x PMC12070536

[45] Fetriyuna F , Purwestri RC , Jati IRAP et al. (2023) Ready-to-use therapeutic/supplementary foods from local food resources: technology accessibility, program effectiveness, and sustainability, a review. Heliyon 9, e22478. 38046154 10.1016/j.heliyon.2023.e22478 PMC10686882

[46] Dickin KL & Seim G (2015) Adapting the Trials of Improved Practices (TIPs) approach to explore the acceptability and feasibility of nutrition and parenting recommendations: what works for low-income families? Matern Child Nutr 11, 897–914. 24028083 10.1111/mcn.12078 PMC6860192

[47] Mostafazadeh-Bora M (2020) The Hawthorne effect in observational studies: threat or opportunity? Infect Control Hosp Epidemiol 41, 491–491. 32052716 10.1017/ice.2020.19

[48] McCarney R , Warner J , Iliffe S et al. (2007) The Hawthorne Effect: a randomised, controlled trial. BMC Med Res Methodol 7, 30. 17608932 10.1186/1471-2288-7-30 PMC1936999

